# PIP degron proteins, substrates of CRL4^Cdt2^, and not PIP boxes, interfere with DNA polymerase η and κ focus formation on UV damage

**DOI:** 10.1093/nar/gkt1400

**Published:** 2014-01-14

**Authors:** Nikolay Tsanov, Chames Kermi, Philippe Coulombe, Siem Van der Laan, Dana Hodroj, Domenico Maiorano

**Affiliations:** ^1^Genome Surveillance and Stability Laboratory, Department of Molecular Bases of Human Diseases, CNRS-UPR1142, Institute of Human Genetics, 141, rue de la cardonille, 34396 Cedex 5, Montpellier, France and ^2^Replication and Genome Dynamics Laboratory, Department of Genome Dynamics, CNRS-UPR1142, Institute of Human Genetics, 141, rue de la cardonille, 34396 Cedex 5, Montpellier, France

## Abstract

Proliferating cell nuclear antigen (PCNA) is a well-known scaffold for many DNA replication and repair proteins, but how the switch between partners is regulated is currently unclear. Interaction with PCNA occurs via a domain known as a PCNA-Interacting Protein motif (PIP box). More recently, an additional specialized PIP box has been described, the « PIP degron », that targets PCNA-interacting proteins for proteasomal degradation via the E3 ubiquitin ligase CRL4^Cdt2^. Here we provide evidence that CRL4^Cdt2^-dependent degradation of PIP degron proteins plays a role in the switch of PCNA partners during the DNA damage response by facilitating accumulation of translesion synthesis DNA polymerases into nuclear foci. We show that expression of a nondegradable PIP degron (Cdt1) impairs both Pol η and Pol κ focus formation on ultraviolet irradiation and reduces cell viability, while canonical PIP box-containing proteins have no effect. Furthermore, we identify PIP degron-containing peptides from several substrates of CRL4^Cdt2^ as efficient inhibitors of Pol η foci formation. By site-directed mutagenesis we show that inhibition depends on a conserved threonine residue that confers high affinity for PCNA-binding. Altogether these findings reveal an important regulative role for the CRL4^Cdt2^ pathway in the switch of PCNA partners on DNA damage.

## INTRODUCTION

Proliferating cell nuclear antigen (PCNA), a processivity factor for replicative DNA polymerases, acts as a docking molecular platform for many factors, and orchestrates several aspects of DNA metabolism such as DNA replication and repair (1). Its homotrimeric ring-shaped structure (2,3) could in theory provide an interaction surface for up to three partners at a time, although binding can be mutually exclusive (4). Binding occurs through a small and highly adaptable PCNA-Interacting Protein motif (PIP box) that tethers partners to a hydrophobic pocket on PCNA (1). To ensure stable interaction, some factors, like the cyclin-dependent kinase inhibitor p21, have evolved a strong binding affinity (5), thus efficiently competing out other factors for binding (5,6).

Other PCNA partners, such as members of the Y-family of translesion synthesis DNA polymerases (TLS pols) that carry out DNA lesions bypass (7), also require an ubiquitin-binding motif that tethers them to an ubiquitin group covalently attached to PCNA (8). Monoubiquitylation of PCNA that occurs on DNA damage, increases the affinity of TLS pol η for PCNA (9–11) and may constitute a mechanism to switch from replicative to TLS pols at stalled replication forks (12). Pol η is recruited at sites of ultraviolet (UV) damage on chromatin to bypass the major UV-induced DNA lesion, the thymine–thymine cyclobutane pyrimidine dimer photoproduct (13,14), and can be visualized by expression of eGFP-tagged Pol η in cells (15). In addition, emerging evidence implicates Y-family TLS pols also in DNA repair (16) outside the S-phase of the cell cycle (17,18). For instance, Pol κ is recruited to UV-damage sites to carry out nucleotide excision repair (NER) (19) in the G1-phase or in quiescent cells (18).

Some PCNA partners are targeted for proteasomal degradation on interaction (20) via polyubiquitylation by the E3 ubiquitin ligase Cullin 4-RING Ligase (CRL4)-Ddb1-Cdt2 (CRL4^Cdt2^). In this reaction, PCNA provides a molecular platform where CRL4^Cdt2^ and the substrate meet (21). Recently, it was discovered that a ‘degron’ module, hereafter called PIP degron, that lies within the PIP box and adjacent amino acids, is essential for degradation (22). Compared with a canonical PIP box (of signature Q/N-x-x-Ψ-x-x-ϑ-ϑ, where Ψ is a hydrophobic residue, mostly M, L, V or I; and ϑ is an aromatic amino acid such as F or Y), a PIP degron contains both a TD motif and a basic amino acid four residues downstream, of signature ‘Q/N-x-x-Ψ-T-D-ϑ-ϑ-x-x-x-R/K’ (22,23). Despite intense investigations, the biological role of this degradation pathway is not completely understood, in particular on DNA damage (20). In metazoans, CLR4^Cdt2^ substrates include replication licensing factor Cdt1 (24,25), p21 and the histone methyltransferase Set8 (26–32). Cdt1 catalyzes loading of the Mcm2-7 helicase at replication origins (33,34) and PCNA-triggered Cdt1 degradation in S-phase prevents re-replication and preserves genome stability (24,35–40). Interestingly, Cdt1 is rapidly proteolysed after DNA damage (within minutes) via the CRL4^Cdt2^ pathway (25,41–43) much faster than during a normal S-phase (28,44–46) by both chromatin-bound PCNA and the SFC^Skp2^ ubiquitin ligase (36,38). Pol η degradation after DNA damage via the CRL4^Cdt2^ pathway in *Caenorabditis elegans*, was proposed as a mechanism to inhibit TLS after completion (47). All these studies implicate CRL4^Cdt2^ in regulating the interaction of PCNA with specific DNA repair and lesion-bypass factors after DNA damage.

We made the hypothesis that CRL4^Cdt2^ may clear PCNA from PIP degron-containing partners to improve accessibility to repair factors. By using the Cdt1 PIP degron, as a tool to test this hypothesis, we provide evidence that PCNA-triggered degradation of Cdt1 is required for efficient eGFP-TLS-Pol η and -Pol κ focus formation on UV damage. By extending this assay to other PIP boxes, we found that this is a specific feature of PIP degrons of Cdt1, p21 and Set8 and not of canonical PIP boxes, like Fen1 or p15(PAF). These results support a model for PCNA partners switch triggered by DNA damage, orchestrated by CRL4^Cdt2^.

## MATERIALS AND METHODS

### Plasmids

Human pEGFP-Pol η (15), pEGFP-Pol κ (48) and pcDNA3-HA-p21^WAF1^ (49) were as described. cDNA clones of human Cdt1, Fen1 and mouse Cullin 4A were purchased from IMAGENES. pcDNA3-Cdt1-HA, pcDNA3-HA-Fen1 and pcDNA3-Cullin 4A-Myc_6_ were generated by polymerase chain reaction (PCR) and cloning into pcDNA3-HA C-terminal, pcDNA3-HA N-terminal and pcDNA3-Myc_6_, respectively, as previously described (50). The deletion mutants Cdt1ΔPIP (Δaa 1–14) and dnCullin4A (Δaa 252–759) were generated by PCR mutagenesis and cloned into pcDNA3-HA C-terminal and pcDNA3-Myc_6_, respectively. To generate Cdt1-PIP^Fen1^ chimera, a *PpuM*I restriction site was created at the 5′ of Cdt1ΔPIP-HA by introducing a silent mutation by PCR. A duplex of annealed oligonucleotides encoding the PIP box of Fen1 with its C-terminal flanking region was inserted into the *Hind*III-*PpuM*I sites to produce pcDNA3-Cdt1-PIP^Fen1^-HA. The same strategy was used to generate Cdt1 mutPIP and Cdt1 R+4A. The myc-PIP box constructs were generated by a similar approach. Annealed oligonucleotides encoding the SV40 large T antigen NLS were inserted into the *Nco*I-*EcoR*I sites of pCS3+MT (51). A second duplex encoding a PIP box with its C-terminal flanking region was subsequently inserted into the *EcoR*I-*Xba*I sites. C-terminally HA-tagged Cdt1 variants were also subcloned into pBabe-puro retroviral vector (52). All constructs were verified by DNA sequencing.

### Cell culture, infection, transfection and electroporation

NIH-3T3, U2OS and Platinum-E (Cell Biolabs) cells were grown in Dulbecco’s modified eagle’s medium supplemented with 10% fetal bovine serum, 2 mM glutamine and antibiotics. For infection, viral particles were generated by transfecting Platinum-E ecotropic packaging cell line with retroviral expression vector (pBabe-puro) encoding Cdt1 variants using homemade PEI reagent. The viral supernatant was diluted (10- to 3000-fold) in normal growth medium to obtain low Cdt1 expression levels. Forty-eight hours after infection, cells were selected in puromycin (2.5 µg/ml)-containing medium. Selected populations were expanded and promptly used for experiments. Cells were transfected with Lipofectamine (Invitrogen). To achieve low Cdt1 expression levels, cells were transfected with pcDNA3-Cdt1-HA and empty vector at 1:20 ratio. For high expression levels, a ratio of Cdt1: empty vector of 3:1 was used. Before electroporation, NIH-3T3 cells were incubated in RPMI-1640 medium for 30 min. After trypsinization, 1 × 10^7 ^cells/ml were resuspended in RPMI-1640 medium and 4 × 10^6 ^cells were mixed with 30 µg of plasmid DNA, and exposed to a single voltage pulse (300 V, 500 µF; Gene-Pulser, Bio-Rad) at room temperature. Electroporated cells were allowed to recover for 5 min in the medium before replating.

### Irradiation

In all experiments, UV-C irradiation at 254 nm was performed with microprocessor-controlled crosslinker (BIO-LINK®) at a dose of 20 J/m^2^ unless stated otherwise.

### Cell viability experiments

Cells were plated at 1.0 × 10^4^ per well in 12-well plates and UV irradiated. Forty-eight hours after irradiation, cell viability was determined using the CellTiter-Glo® Luminescent Cell Viability assay (Promega).

### Antibodies

ORC2 antibody was from Marcel Méchali (IGH, Montpellier). Pol η, Mcm2 (AbCam), PCNA and β-actin (Sigma), HA (Y-11, Santa Cruz Biotechnology), Myc9B11, PR-Set/Set8, P-p53 (Ser15), P-H2AX (Ser139; Cell Signalling) and Cdt1 (Millipore).

### Immunofluorescence and microscopy

Cells were grown on coverslips before co-transfection. Four hours after UV-C irradiation, cells were fixed with 3.2% paraformaldehyde for 15 min at room temperature, washed three times with phosphate buffered saline (PBS) and permeabilized with 0.2% Triton X-100 for 5 min on ice. To detect Cdt1-HA variants or myc-PIP box peptides, cells were blocked with PBS + 3% bovine serum albumin (BSA) for 30 min and incubated with anti-HA or anti-myc primary antibodies, respectively, for 1 h at room temperature. Cells were washed twice with PBS + 3% BSA and incubated with Texas Red-conjugated donkey anti-rabbit IgG (Jackson Immunoresearch) for 1 h at room temperature. After washing twice with PBS + 3% BSA, cells were mounted with ProlongGold DAPI (Invitrogen). eGFP-Pol η or eGFP-Pol κ foci were analyzed with Leica DM6000 epifluorescence microscope (RIO imaging facility). Images were acquired using a Coolsnap HQ CCD camera (Photometrics) and metamorph software (Molecular Devices).

### Foci formation assay

Cells were co-transfected with eGFP-Pol η or eGFP-Pol κ and Cdt1 variants, p21, Fen1 or myc-PIP box constructs and incubated for 24 h before UV-C irradiation. Four hours after irradiation, cells were fixed, washed three times with PBS and mounted with Prolong Gold DAPI (Invitrogen). The percentage of eGFP-Pol η- or eGFP-Pol κ-expressing cells displaying eGFP-Pol η or eGFP-Pol κ foci was determined by scoring at least 200 nuclei for each condition. Nuclei containing <30 foci were scored as negatives. Means and standard deviation (error bars) of three independent experiments are shown.

### Cell lysis and immunoprecipitation experiments

Co-immunoprecipitations were performed as described (50). Briefly, cells were rinsed once in PBS and incubated with ice-cold lysis buffer (50 mM Tris–HCl, pH 7.4, 100 mM NaCl, 50 mM NaF, 5 mM EDTA, 40 mM β-glycero-phosphate, 1% Triton X-100 and protease inhibitors) for 30 min on ice before scraping. Equivalent amounts of protein were incubated for 4 h at 4°C with HA-coupled protein A agarose beads (Roche). After extensive washing with lysis buffer, bound proteins were eluted in Laemmli buffer. Alternatively, after cell lysis, whole cell extracts were clarified by centrifugation at 16 000*g* for 10 min at 4°C. Protein concentration of the clarified lysates was estimated using BCA method (Pierce).

### Chromatin isolation

Chromatin-enriched and soluble fractions were prepared using CSK-extraction procedure. Briefly, cell pellets were lysed in CSK buffer (10 mM PIPES, pH 6.8, 100 mM NaCl, 300 mM sucrose, 1 mM EGTA, 1 mM MgCl_2_, 0.5 mM DTT, 1 mM ATP, 0.2% Triton X-100 and protease inhibitors) for 10 min on ice. After centrifugation at 800*g* for 3 min at 4°C, the supernatant (Triton-soluble fraction) was recovered. The pellet (Triton-insoluble fraction) was resuspended in CSK buffer and incubated for 10 min on ice. After centrifugation, the pellet (chromatin-enriched fraction) was lysed in Laemmli Buffer.

### UV-induced cell death assay

This assay was performed as previously described (53). Briefly, cells were electroporated with Cdt1 variants or myc-PIP box^Cdt1^ constructs and incubated for 24 h before 10 J/m^2^ UV-C irradiation. Twenty-four hours after irradiation, cells were harvested, washed twice in PBS and fixed in ice-cold 70% ethanol at −20°C overnight. Thawed cells were washed twice in PBS and incubated with 50 µg/ml RNase A at 37°C for 1 h. DNA was stained with propidium iodide (25 µg/ml). Cells were analyzed with a FACScalibur flow cytometer using CellQuestPro software. The percentage of cells displaying a DNA content lower than 2C was assessed.

## RESULTS

### CRL4^Cdt2^-mediated proteolysis facilitates UV-induced eGFP Pol η and Pol κ focus formation

On irradiation of cells with UV light, Pol η is recruited to chromatin by interaction with PCNA and accumulates in discrete, microscopically visible foci that co-localize with sites of UV damage, visualized on expression of Pol η fused to enhanced green fluorescent protein (eGFP-Pol η). Importantly, eGFP-Pol η restores the UV sensitivity of cells mutated in the Pol η gene (XP-V mutant) and therefore constitutes a useful marker of translesion DNA synthesis *in vivo* (15).

We sought to determine whether failure to degrade CRL4^Cdt2^ substrates on DNA damage might impact on eGFP-Pol η foci formation. We first treated cells with the proteasome inhibitor MG132 to stabilize all CRL4^Cdt2^ substrates after UV damage (25,27), and observed a strong decrease in the number of cells with eGFP-Pol η foci (Supplementary Figure S1A). Next, we disrupted the CRL4^Cdt2^ pathway by expressing a dominant negative mutant of Cullin 4A (54), a scaffold for CRL4^Cdt2^ complexes (Supplementary Figure S1B–E), and obtained a similar result. These results show that ubiquitin-dependent proteasomal degradation is a prerequisite for eGFP-Pol η focus formation, thus implicating the CRL4^Cdt2^ pathway in this regulation.

**Figure 1.**
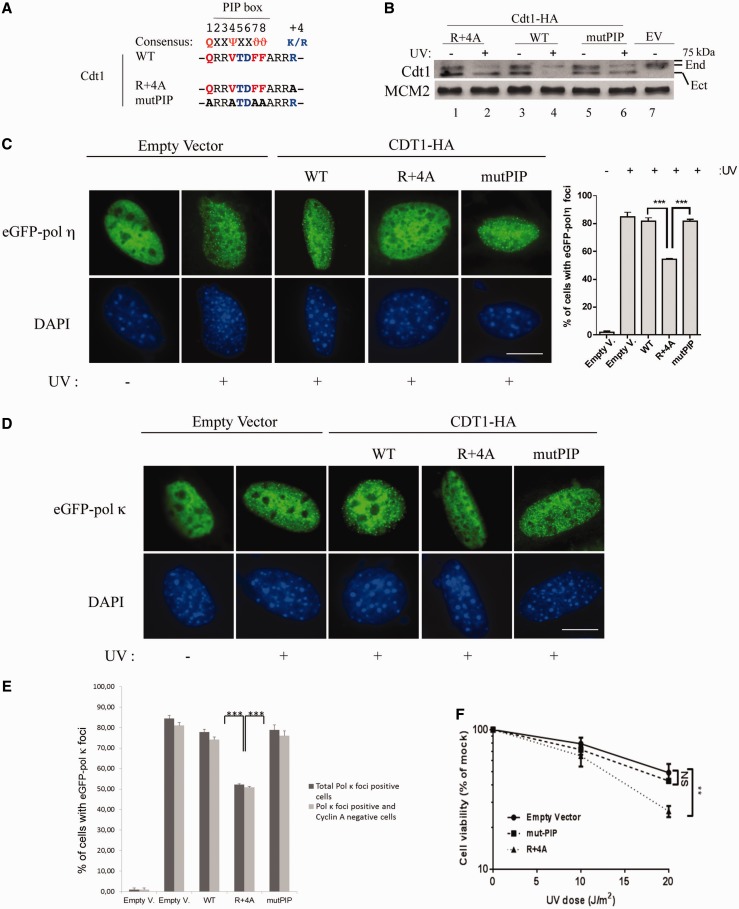
The Cdt1^R+4A^ mutant affects UV-induced eGFP-Pol η and κ focus formation. (**A**) Sequence comparison between Cdt1 WT PIP box, the degron mutant R+4A and the PIP box mutant mutPIP. The residues essential for interaction with PCNA are shown in red (or light gray), and the residues that define the PIP degron are shown in blue (or dark gray). Ψ = Val, Leu, Ile, Met; ϑ = Tyr, Phe. (**B**) The basic residue (R) four amino acids downstream (+4) of the Cdt1 PIP box is required for UV-induced Cdt1 degradation. NIH-3T3 cells were transduced with pBabe-puro retroviral vector encoding either HA-tagged Cdt1^WT^, Cdt1^R+4A^, Cdt1^mutPIP^ or empty vector (EV) and selected with puromycin for 2 days. Cell lysates were prepared 4 h after mock (−) or UV (+) irradiation and analyzed by western blot to detect both endogenous (End) and ectopically expressed human Cdt1(Ect), that is smaller in size than the mouse orthologue. (**C** and **D**) NIH-3T3 cells transduced as in (B) were subsequently transfected with eGFP-Pol η or eGFP-Pol κ (panel D) and irradiated as in (B). Control cells were transduced with empty vector. Four hours after irradiation, the distribution of eGFP-Pol η or -Pol κ was examined by fluorescence after fixation. Scale bar: 10 µm. (Right panel) The percentage of eGFP-Pol η-expressing cells in which Pol η was localized in nuclear foci was assessed. Means and standard deviation of three independent experiments are shown. ***P < 0.0001. (**E**) The percentage of eGFP-Pol κ-expressing cells in which Pol κ was localized in nuclear foci (black bars) that also stained negative for Cyclin A (gray bars) was assessed. Means and standard deviation of three independent experiments are shown. ***P < 0.0001. (**F**) Cell viability of cells transduced as in (B) with Cdt1^R+4A^, Cdt1^mutPIP^ or empty vector. **P < 0.01 (n = 3). NS, nonsignificative.

Next, we determined to which extent partial inhibition of CRL4^Cdt2^-dependent proteolysis affects eGFP-Pol η focus formation compared with inhibition of Cullin 4A. For this purpose, we generated two nondegradable mutants of the CRL4^Cdt2^ substrate Cdt1, described in Figure 1A. The first one (R+4A mutant) binds PCNA but cannot be degraded (22), since it is mutated in the PIP degron by alanine substitution (A) of the basic residue (R) essential for CRL4^Cdt2^-dependent degradation, four amino acids (+4) downstream of the PIP box. The second is mutated in the PIP box (mut PIP) and therefore cannot interact with PCNA (21). NIH-3T3 cells were transduced with retroviral vectors encoding HA-tagged Cdt1 variants expressed to similar levels than endogenous Cdt1 to perform the experiments described below (Figure 1B). Consistent with previous reports (22,23), Cdt1^R+4A^ remains stable on UV damage, as the Cdt1^mutPIP^, whereas Cdt1 wild-type (WT) and endogenous Cdt1 are efficiently degraded (Figure 1B). Importantly, eGFP-Pol η foci formation was also reduced in cells expressing the Cdt1^R+4A^ mutant compared with cells expressing Cdt1^WT^ (Figure 1C), while Cdt1^mutPIP^ did not have a significant effect.

**Figure 2.**
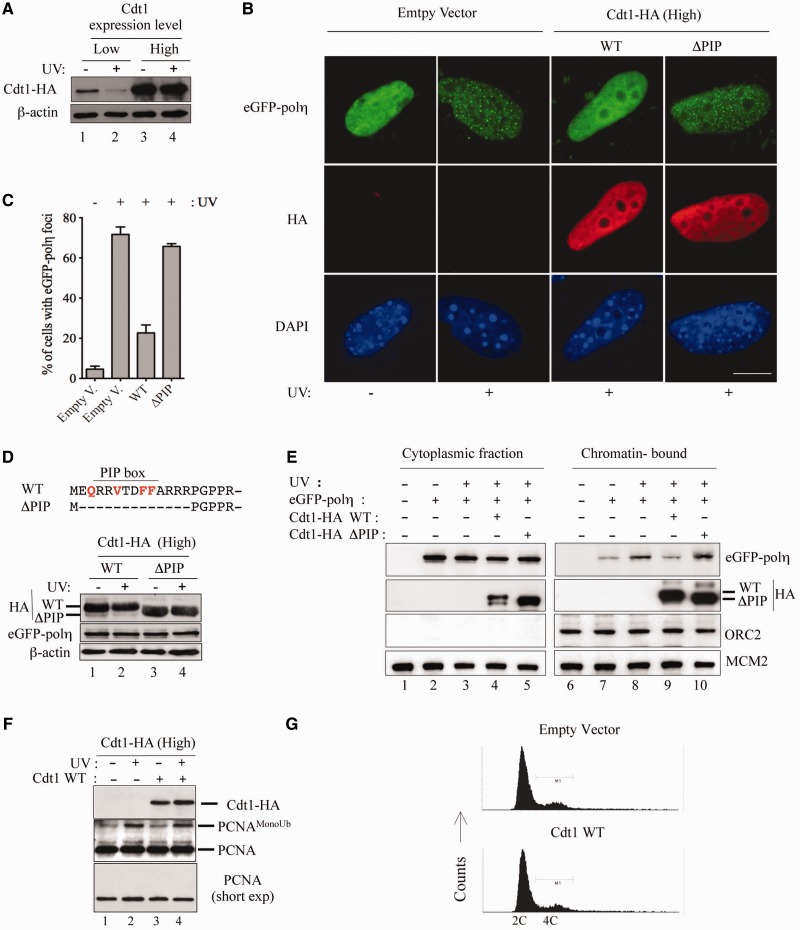
Overexpression of Cdt1 impairs eGFP-Pol η focus formation after UV irradiation, in a PIP box-dependent fashion. (**A**) Western blot of NIH-3T3 cells transiently transfected with pcDNA3^(strong promoter)^ vector encoding Cdt1-HA WT as indicated in ‘Materials and Methods’ section. Cells were UV irradiated (+) or mock irradiated (−) 24 h after transfection. Four hours later, cells were harvested and processed for immunoblotting with antibodies to HA, or β-actin. (**B**) NIH-3T3 cells were co-transfected with eGFP-Pol η and either pcDNA3 vector encoding Cdt1-HA^WT^, Cdt1-HA^ΔPIP^ or empty vector and UV irradiated as in (A). Four hours after irradiation, cells were fixed and stained with anti-HA antibody. The distribution of eGFP-Pol η was detected by GFP fluorescence (green), and the staining of Cdt1-HA (red) obtained by indirect immunofluorescence. Scale bar: 10 µm. (**C**) Quantification of the data shown in (B). Means and standard deviation of three independent experiments are shown. (**D**) Western blot of NIH-3T3 cells co-transfected with eGFP-Pol η and the indicated constructs and irradiated as in (B). The sequence of Cdt1ΔPIP is shown (dotted line represents deleted residues). The residues essential for interaction with PCNA are shown in red (or light gray). (**E**) Cdt1 inhibits PCNA-mediated recruitment of Pol η to chromatin after UV irradiation. UV-induced binding of Pol η to chromatin. U2OS cells were transfected with the indicated plasmids. Cells were detergent-extracted 4 h after mock (−) or UV (+) irradiation. The distributions of Pol η, Cdt1^WT^ and Cdt1^ΔPIP^ in the cytoplasmic and chromatin-enriched fractions are shown. (**F**) Monoubiquitylation of PCNA is not affected by Cdt1 expression. NIH-3T3 were selected with puromycin after co-transfection with pBabe-puro and either Cdt1-HA^WT^ (+) or pcDNA3 empty vector (−). Cells were irradiated as in (A) and the expression level of Cdt1-HA and PCNA monoubiquitylation were analyzed by western blot. A low exposure of nonubiquitylated PCNA is also shown (short exp). (**G**) Overexpression of Cdt1 in NIH-3T3 cells does not affect cell cycle. Three days following transfection with Cdt1^WT^ or empty vector, cells were fixed and stained with propidium iodide, and processed for FACS analysis.

We also analyzed formation of eGFP-Pol κ foci, another PIP box-containing TLS polymerase also implicated in NER in the G1-phase of the cell cycle. We observed that on UV damage, eGFP-Pol κ focus formation occurred mainly in cells that stained negative for Cyclin A (Figure 1E, compare black bars with gray bars, and Supplementary Figure S2A) in line with previous observations (19,55). Importantly, we observed inhibition of eGFP-Pol κ focus formation by the Cdt1^R+4A^ mutant (Figure 1D and E, R+4A), unveiling the DNA damage-dependent contribution of CRL4^Cdt2^ in G1-phase, when the S-phase CRL4^Cdt2^ pathway is off (20). Importantly, the percent of Cyclin A-negative cells (>60% of the total cell population), did not change in cells expressing the different Cdt1 variants (Supplementary Figure S2B), consistent with the cell cycle distribution of NIH-3T3 cells (Figure 2G). These results are consistent with a previous report showing that UV-dependent Pol κ focus formation is excluded from S-phase cells (18). Of note, expression of nondegradable Cdt1 (R+4A mutant) at endogenous levels inhibits both eGFP-Pol η and -Pol κ focus formation, to a lower extent compared with treatment with the proteasome inhibitor MG132, or to Cullin 4A inhibition. These later induce global stabilization of CRL4^Cdt2^ substrates and therefore interfere with TLS polymerases foci formation to a greater extent. Because Cdt1^mutPIP^ did not significantly interfere with either eGFP-Pol η or -Pol κ focus formation (Figure 1), it suggests that interference depends on interaction with PCNA. Importantly, the Cdt1^R+4A^ mutant also reduced cell viability on UV irradiation (Figure 1F) that was not further affected on downregulation of Pol κ expression (Supplementary Figure S2C and D), as expected if the Cdt1^R+4A^ mutant interferes with TLS function.

Altogether, these results show that persistence of the Cdt1^R+4A^ nondegradable substrate of CRL4^Cdt2^ on UV irradiation interferes with the function of at least two TLS polymerases (η and κ) involved in distinct repair pathways.

### High levels of Cdt1^WT^, and not of Cdt1^ΔPIP^, impair formation of eGFP-Pol η foci

We next determined whether overriding the CRL4^Cdt2^ degradation pathway may have a stronger effect than the Cdt1^R+4A^ mutant on eGFP-Pol η focus formation. To this end, we expressed Cdt1^WT^ at levels that override its degradation, and analyze eGFP-Pol η focus formation after UV damage. Ectopic Cdt1^WT^, expressed in NIH-3T3 cells under control of a strong promoter (CMV), was not degraded on UV irradiation (Figure 2A, lanes 3–4), while degradation was still observed at a lower expression level (lanes 1–2). Analysis of eGFP-Pol η accumulation (Figure 2B) shows that UV-induced Pol η focus formation was affected in cells expressing high levels of Cdt1. Quantification shows that the number of Pol η foci-forming cells now dropped to 22% on expression of Cdt1^WT^ compared with 72% in control cells (Figure 2C). A similar result was obtained by analysis of eGFP-Pol κ, and a Cdt1 mutant lacking the PCNA interaction motif (Cdt1^ΔPIP^) did not have a significant effect (Supplementary Figure S3A and B). This mutant localized into the nucleus (Figure 2B, ΔPIP, HA) was expressed at a similar level as Cdt1^WT^ (panel D), and was also chromatin-bound (Supplementary Figure S4A), consistent with a previous report (56). This is expected because Cdt1 chromatin binding also depends on the Origin Recognition Complex, and the Cdt1 N-terminus, which contains the PIP box, is dispensable for DNA replication (34).

We also observed impaired UV-induced Pol η foci formation on expression of Cdt1 in U2OS human cells (Supplementary Figure S4B). eGFP-Pol η is enriched in the chromatin fraction specifically on UV irradiation (Figure 2E, lanes 7–8), and its recruitment is impaired on expression of Cdt1^WT^, and not of Cdt1^ΔPIP^ (lanes 9–10). Finally, expression of Cdt1 mutated in the key residues essential for PCNA interaction (Cdt1^mutPIP^) did not affect eGFP-Pol η foci formation (Supplementary Figure S4C). Taken together, these results show that Cdt1^mutPIP^ loses the potential to inhibit recruitment of Pol η to chromatin and to impair Pol η foci formation on UV irradiation. We also verified that high Cdt1^WT^ expression did not impair UV-induced PCNA monoubiquitylation (Figure 2F), essential for Pol η recruitment to damage sites, nor induce global cell cycle changes (Figure 2G). These observations rule out the possibility that Cdt1^WT^ overexpression may affect Pol η focus formation indirectly via interference with PCNA monoubiquitylation, or via an indirect cell cycle effect, and also show that PCNA is still competent to be posttranslationally modified.

In mammalian cells, expression of Cdt1 at high levels promotes DNA rereplication only in certain cell lines (57,58). Consistent with this observation, we observed that expression of Cdt1 at high levels induces rereplication in human U2OS cells, and not in mouse NIH-3T3 cells (Supplementary Figure S4D); however, interference with UV-induced eGFP-Pol η foci was observed in both cell lines (Figure 2B and C and Supplementary Figure S4B), suggesting that the ability of Cdt1 to interfere with Pol η focus formation is independent from its function in DNA replication.

### The Cdt1 PIP box is sufficient to impair Pol η foci formation and to induce UV-dependent cell death

**Figure 3.**
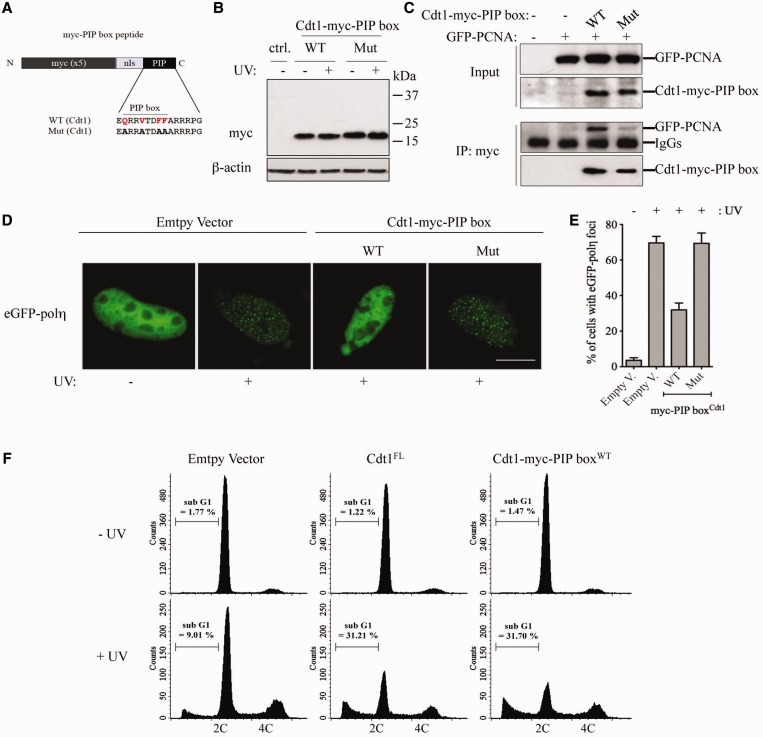
Cdt1 PIP box-peptide impairs Pol η focus formation and induces UV-dependent cell death. (**A**) Description of Cdt1-myc-PIP box constructs obtained by fusion of a c-myc tag, SV40 large T-antigen nuclear localization signal (nls) and a PIP box (PIP). The sequence used to generate WT or mutant (Mut) PIP box of Cdt1 is shown. (**B**) Western blot of NIH-3T3 cells transfected with Cdt1-myc-PIP box^WT^ or Cdt1-myc-PIP box^Mut^, or empty vector (ctrl.). Transfected cells were mock (−) or UV irradiated (+), and 4 h later were processed for immunoblotting with antibodies to myc or β-actin. (**C**) The Cdt1-myc-PIP box^WT^ interacts specifically with PCNA. NIH-3T3 cells were co-transfected with eGFP-PCNA and either the indicated Cdt1-myc-PIP box constructs or empty vector (−). After 24 h, cell lysates were prepared, immunoprecipitated with an antibody against myc and immunoblotted with antibodies to PCNA or myc. (**D** and **E**) NIH-3T3 cells were co-transfected with eGFP-Pol η and either the indicated Cdt1-myc-PIP box constructs or empty vector. Transfected cells were irradiated as in (B) and eGFP- Pol η foci were examined 4 h later (D). The percentage of eGFP-Pol η-expressing cells displaying foci was assessed (E). Means and standard deviation of three independent experiments are shown. Scale bar: 10 µm. (**F**) The Cdt1-myc-PIP box induces UV-dependent cell death. NIH-3T3 cells were electroporated with full-length (FL) Cdt1-HA, Cdt1-myc-PIP box or empty vector. Twenty-four hours after transfection, cells were mock or UV irradiated with 10 J/m^2^, fixed and stained 24 h later with propidium iodide and processed for FACS analysis. The percentage of sub-G1 cells was assessed.

To determine whether the PIP box of Cdt1 on its own affects Pol η focus formation, we generated and expressed only the Cdt1^WT^ PIP box, or a mutant that cannot interact with PCNA (Cdt1^Mut^), fused to the c-myc epitope and the SV40 nuclear localization signal to facilitate nuclear retention (Cdt1-myc-PIP box, Figure 3A and B). We verified by immunoprecipitation that Cdt1-myc-PIP box^WT^ and not Cdt1-myc-PIP box^Mut^ interacts specifically with PCNA (Figure 3C). Strikingly, we observed that expression of Cdt1-myc-PIP box^WT^ impaired UV-induced Pol η focus formation, while the mutant Cdt1-myc-PIP box^Mut^ that cannot interact with PCNA showed no inhibition (Figure 3D and E), similar to what observed with full-length Cdt1. Similarly, Cdt1-myc-PIP box^WT^ severely impaired formation of UV-induced Pol κ foci, while Cdt1-myc-PIP box^Mut^ showed no inhibition (Supplementary Figure S3C). Notably, both full-length Cdt1 and Cdt1-myc-PIP box^WT^ impaired UV-induced Pol κ foci formation more efficiently than Pol η foci (compare Supplementary Figure S3B with Figure 2C, and Supplementary Figure S3C with Figure 3E), consistent with the lower PCNA-binding affinity of the Pol κ PIP box compared with that of Pol η (59).

Upon UV damage, Pol η is essential for cell viability (15). Hence, if expression of Cdt1 interferes with TLS (as well with other DNA repair pathways, see ‘Discussion’ section) it is expected that cell death should increase specifically only on UV irradiation. This was previously shown to be the case for p21 (60). To address this question, we quantified the formation of hypodiploid (sub-G1) cells after UV irradiation as previously described (53). Consistent with data shown in Figure 1F, we observed >3-fold increase of UV-specific sub-G1 cells on expression of Cdt1^WT^ and not Cdt1^mutPIP^ (Figure 3F and Supplementary Figure S4E). In addition, expression of Cdt1-myc-PIP box^WT^ also induced UV-dependent cell death to a similar level than full-length Cdt1 (FL, Figure 3F). Importantly, neither Cdt1^FL^ nor Cdt1-myc-PIP box induced cell death in the absence of UV damage, but specifically affected cell viability only on DNA damage, suggesting interference with TLS function and/or other repair pathways.

Collectively, these results demonstrate that the Cdt1 PIP box on its own is both required and sufficient to inhibit UV-induced Pol η foci and to induce UV-dependent cell death.

### PIP degrons of Cdt1, p21 and Set8 are potent inhibitors of UV-induced Pol η foci

**Figure 4.**
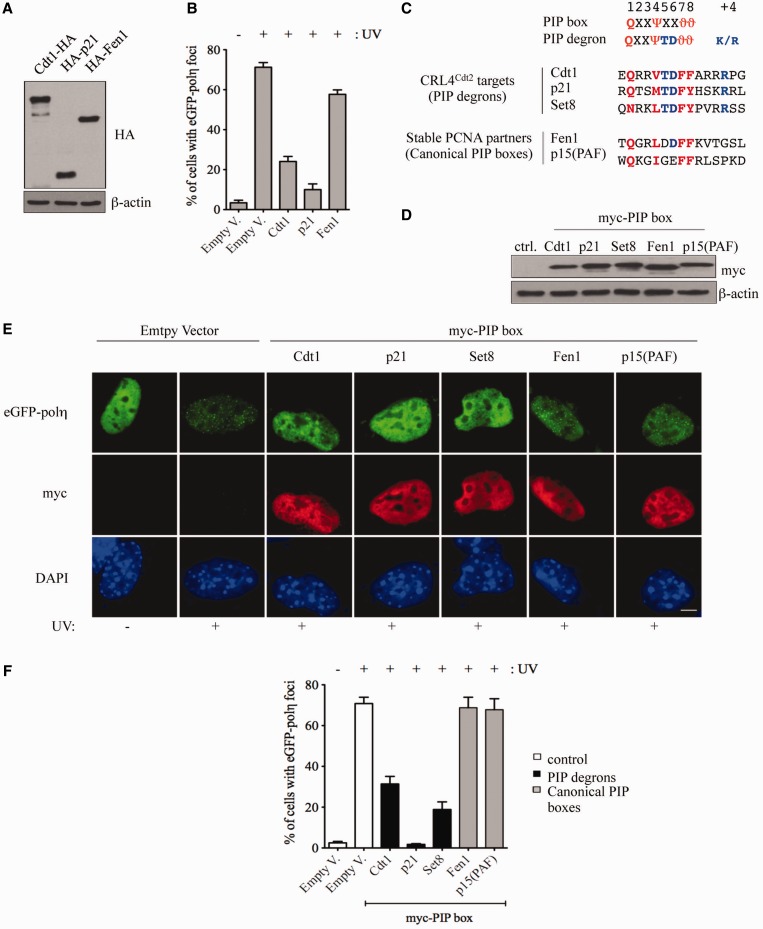
PIP degron peptides derived from CRL4^Cdt2^ targets inhibit accumulation of Pol η into UV-damage foci. (**A**) NIH-3T3 cells transfected with pcDNA3 vector encoding either HA-tagged full-length Cdt1, p21 or Fen1 were analyzed by western blot with antibodies specific for HA or β-actin. (**B**) NIH-3T3 cells were co-transfected with eGFP-Pol η and the indicated constructs. The percentage of eGFP-Pol η-expressing cells displaying Pol η foci was determined 4 h after mock (−) or UV (+) irradiation. Means and standard deviation of three independent experiments are shown. (**C**) Sequences used to generate myc-PIP box peptides derived from CRL4^Cdt2^ substrates (PIP degrons) or stable PCNA partners (canonical PIP boxes). The residues essential for interaction with PCNA are shown in red (or light gray), residues that define the PIP degron are shown in blue (or dark gray). (**D**) Western blot of NIH-3T3 cells transfected with either the indicated myc-PIP box constructs, described in (C), or empty vector (ctrl.). Cell lysates were immunoblotted with antibodies specific to myc or β-actin. (**E** and **F**) Comparison of the potential of PIP degron- and canonical PIP box-containing peptides to inhibit Pol η focus formation. NIH-3T3 cells were co-transfected with eGFP-Pol η and the indicated myc-PIP box constructs and irradiated as in (B). Four hours later, cells were fixed and stained with anti-myc antibody and observed by fluorescence microscopy (E). The percentage of eGFP-Pol η-expressing cells in which Pol η accumulates in nuclear foci was assessed (F). The distribution of eGFP-Pol η (green) and the staining of myc-PIP box peptides (red) in the same cell are shown (E). Scale bar: 5µm.

As we observe for Cdt1, it was previously shown that p21 overexpression can also interfere with UV-induced Pol η foci formation via its PIP box (60). This raises the question of whether this is a general property of all PIP box-containing proteins (61). To address this question, we compared the ability of Cdt1, p21 as well as another PIP box-containing protein, the flap endonuclease-1 Fen1, to impair formation of eGFP-Pol η foci after UV-induced damage. We observed that when expressed at similar levels (Figure 4A), only Cdt1 and p21 could interfere with formation of eGFP-Pol η foci, while Fen1 only had a marginal effect (Figure 4B), consistent with a previous report showing that the PCNA-binding affinity of p21 is 725-fold higher than that of Fen1 (5).

To extend our analysis to other PCNA-binding proteins, we used the same strategy described in Figure 3A, to create a series of myc-PIP box constructs (Figure 4C) belonging to two distinct categories, (i) substrates of CRL4^Cdt2^ (PIP degrons) and (ii) canonical PIP boxes. These constructs were expressed at similar levels in NIH-3T3 cells (Figure 4D), and they all localized in the nucleus (panel E). Consistent with the weak inhibition of Pol η foci formation observed by expression of Fen1 (panel B), the expression of either myc-PIP box^Fen1^ or myc-PIP box^p15(PAF)^ did not impair the assembly of Pol η foci following UV irradiation (panels E–F). In contrast, PIP degrons such as Cdt1, p21 and Set8 showed strong inhibitory activity (panel F). These results suggest that PIP degrons, rather than canonical PIP boxes, can efficiently compete for PCNA binding, although it cannot be excluded that exceptions to this rule may exist.

### The conserved TD motif of PIP degrons is critical for interference with Pol η focus formation

**Figure 5.**
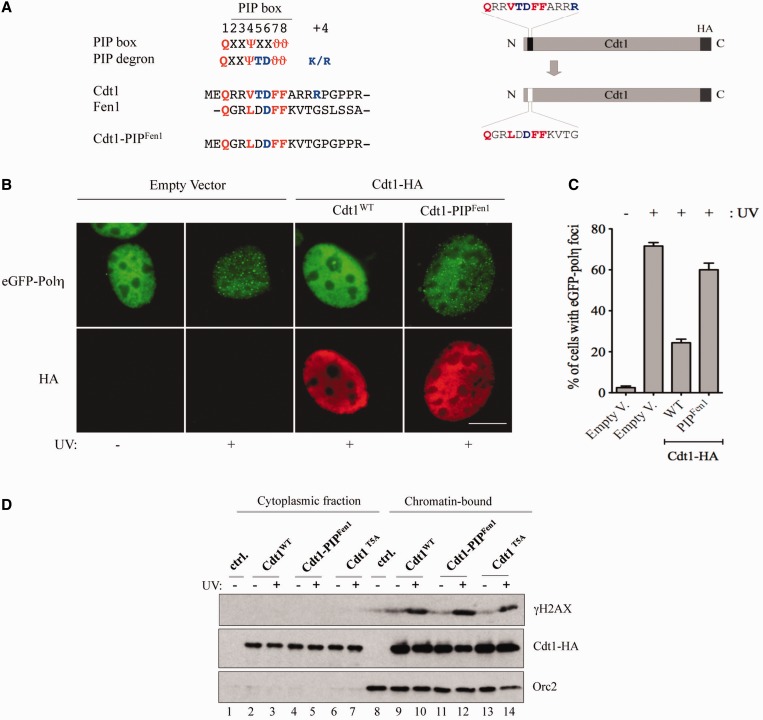
The Fen1 PIP box does not interfere with eGFP-Pol η focus formation. (**A**) Comparison of the sequence of the Cdt1 PIP box with that of Fen1 (left panel). Description of Cdt1-PIP^Fen1^ chimeric fusion (right panel). (**B**) The Cdt1-PIP^Fen1^ chimera does not interfere with UV-induced Pol η foci. NIH-3T3 cells were co-transfected with eGFP-Pol η and either the indicated constructs or empty vector. Four hours after mock (−) or UV irradiation (+), cells were fixed and immunostained with anti-HA antibody. The distribution of eGFP-Pol η (green) and the staining of Cdt1-HA (red) in the same cell are shown. Scale bar: 10 µm. (**C**) The percentage of eGFP-Pol η-expressing cells displaying Pol η foci was determined. Means and standard deviation of three independent experiments are shown. (**D**) Subcellular fractionation of NIH-3T3 cells transfected with the indicated Cdt1 variants or empty vector (ctrl.). Four hours after mock (−) or UV irradiation, cells were lysed and fractionated into soluble (cytoplasmic) and insoluble (chromatin-bound) fractions. Extracts were analyzed by western blot with the indicated antibodies. Activation of the DNA damage checkpoint was monitored with an anti-phospho-specific H2AX antibody (γH2AX).

To address whether interference with eGFP-Pol η focus formation is a specific feature of a PIP degron, we exchanged the PIP degron of Cdt1 with the canonical PIP box of Fen1 (Figure 5A) and tested the ability of the resulting chimeric protein Cdt1-PIP^Fen1^ to interfere with Pol η accumulation into foci. Interestingly, we observed that the ability of Cdt1 to impair Pol η focus formation after UV damage was strongly reduced when its PIP degron was exchanged with the PIP box of Fen1 (Figure 5B–C), and was comparable with that observed with Cdt1 lacking its PIP box (Cdt1^ΔPIP^), shown in Figure 2B–C. Importantly, Cdt1-PIP^Fen1^ mutant was expressed at a similar level as Cdt1^WT^ and was chromatin bound (Figure 5D).

**Figure 6.**
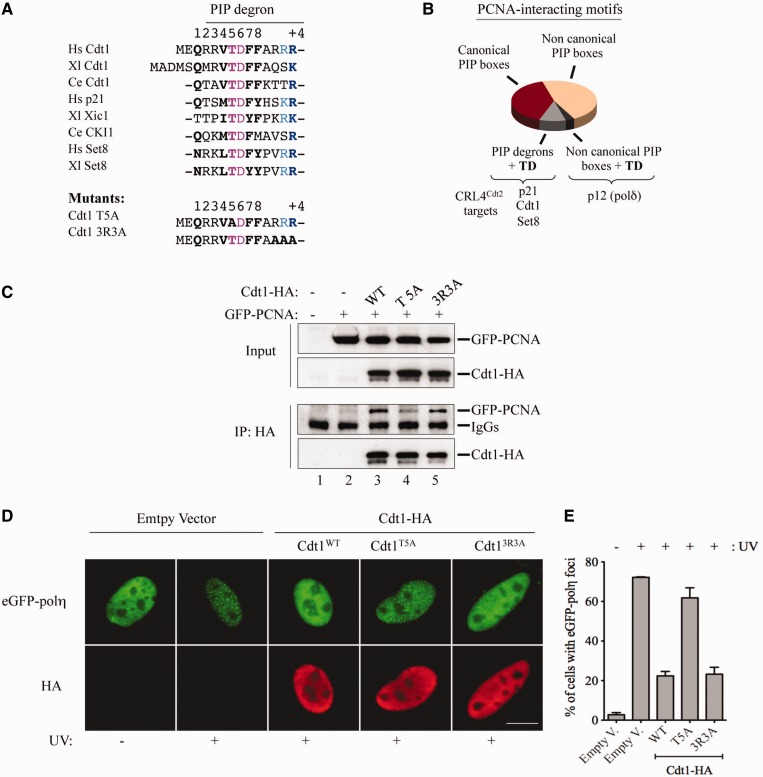
The threonine (T5) within Cdt1 PIP degron confers high-affinity PCNA-binding and is critical for inhibition of Pol η foci. (**A**) Conservation of the threonine residue (T5) in PIP degrons. The sequences of Cdt1^T5A^ and Cdt1^3R3A^ mutants are shown. (**B**) Presence of the TD motif within PCNA-interacting motifs. The PIP boxes of thirty known partners of PCNA in humans have been organized in several categories: canonical PIP boxes (consensus), noncanonical PIP boxes (differ from consensus) and PIP degrons. Those containing a TD motif within their PIP box are indicated (See also Supplementary Figure S5). (**C**) Mutation of the threonine residue (T5) to alanine strongly reduces Cdt1 binding to PCNA. NIH-3T3 cells were co-transfected with eGFP-PCNA and pcDNA3 vector encoding either WT Cdt1^WT^ or Cdt1 mutants described in panel (A). After 24 h, cell lysates were prepared and immunoprecipitated with an antibody against HA, and immunoblotted with antibodies to PCNA and HA. (**D**) NIH-3T3 cells were co-transfected with eGFP-Pol η and either the indicated constructs or empty vector. Four hours after mock (−) or UV irradiation (+), cells were fixed and immunostained with anti-HA antibody. Scale bar: 10 µm. (**E**) Quantification of the data shown in (D). Means and standard deviation of three independent experiments are shown.

Next, we sought to identify the residues within the Cdt1 PIP degron, which confer its ability to impair UV-induced Pol η focus formation. PIP degrons differ from canonical PIP boxes in that they contain two highly conserved motifs (Figure 6A), (i) a TD motif and (ii) adjacent basic residues required for CRL4^Cdt2^-dependent degradation (22,23,44). The TD motif confers high-affinity PCNA-binding (22,23) and is absent in the Cdt1-PIP^Fen1^ chimera (Figure 5A) as well as in the vast majority of PCNA-binding proteins (Figure 6B and Supplementary Figure S5A), suggesting that it may be important for interference with Pol η focus formation. To test this possibility, we individually disrupted either the TD motif of Cdt1 by substitution of the threonine residue by alanine (Cdt1^T5A^), or, as a control, we mutated the basic motif downstream of its PIP box by replacing all basic residues by alanine (Cdt1^3R3A^), as pictured in Figure 6A. Immunoprecipitation experiments show that both Cdt1^WT^ and Cdt1^3R3A^ efficiently interact with PCNA (Figure 6C, IP: HA, lanes 3 and 5), whereas the Cdt1^T5A^ mutant has a much weaker PCNA-binding affinity (lane 4), confirming previous results (22,23). We observed that the Cdt1^3R3A^ mutant impaired the accumulation of Pol η into foci in a similar way as to Cdt1^WT^ (Figure 6D and E), indicating that the basic (RRR) motif is not required for inhibition of Pol η focus formation. In contrast, the Cdt1^T5A^ mutant only weakly affected Pol η foci formation, although it bound chromatin with similar efficiency as Cdt1^WT^ (Supplementary Figure S5D, compare lanes 9–10 with lanes 13–14).

Collectively, these results show that a point mutation within Cdt1 PIP degron considerably reduces both its affinity for PCNA and the potential to impair Pol η foci formation. We conclude that the threonine residue within the Cdt1 PIP degron, which is conserved among PIP degrons, is a critical residue for interference with Pol η focus formation after UV irradiation.

## DISCUSSION

In this work, we have provided evidence that activation of CRL4^Cdt2^ by DNA damage facilitates access of specific repair factors to PCNA, such as TLS Pol η and Pol κ, showing that CRL4^Cdt2^ plays an additional role in TLS DNA synthesis, independent of PCNA monoubiquitylation (62) involving efficient clearing of PIP degrons from PCNA on UV damage. This regulation may ensure that PIP degrons, which have a stronger affinity for PCNA than canonical PIP boxes, are efficiently removed because their presence may be deleterious for the cell at a given time. For example, it was shown that p21 interferes with TLS activity *in vivo* (63), as well as with TLS pol η focus formation (60) via its PIP box. Moreover, removal of chromatin-bound Cdt1 in early S-phase constitutes one mechanism to limit replication to only one round per cell cycle. On DNA damage, Cdt1 is degraded within minutes (25,35), but the biological significance of this instant degradation has been so far elusive. Because Cdt1 has no further role in replication after MCM2-7 recruitment (64), its degradation may be important to avoid reinitiation at just fired origins, or alternatively, to facilitate TLS-dependent replication fork restart. Under our experimental conditions, we could not observe rereplication on stabilization of Cdt1 after UV damage, nor hyperactivation of the DNA damage checkpoint, a molecular sign of abnormal replication (58,65), suggesting that removal of Cdt1 after damage may have no roles in repressing reinitiation of DNA synthesis. In contrast, our data indicate that expression of the Cdt1 PIP degron on its own affects cell viability on UV damage, similar to full-length Cdt1, suggesting interference with TLS function, independently from Cdt1 function in DNA replication. There is evidence that replication fork restart at UV lesions requires Pol η (66), which may suggest that removal of Cdt1 from PCNA after UV damage in early S-phase may facilitate Pol η recruitment to reduce replication stress. This interpretation is also consistent with a previous report showing that Pol η is essential for cell viability on UV damage (15), and that disruption of its PCNA interaction region strongly affects cell viability (67). In support of this possibility, recent data show that proteolysis of chromatin-bound FBH1 helicase (another PIP degron) is important to maintain genomic stability by preventing homologous recombination after replication stress (68,69) and facilitate lesion bypass (70). Moreover, p21 degradation has been now shown to be important in replication bypass at forks stalled by UV lesions (71). Finally, recent data indicate that activation of CRL4^Cdt2^ after DNA damage may be also important to stall ongoing DNA synthesis by degradation of the p12 subunit of DNA polymerase δ (72).

The experimental data provided in our work suggest that regulated proteolysis of PCNA cofactors may represent a way to regulate specific interactions, an attractive model previously proposed as mechanism to increase PCNA accessibility (31,61,63), in addition to reversible posttranslational modifications of PCNA such as ubiquitylation and sumoylation (1).

### Possible role of PCNA-dependent degradation in DNA repair

New emerging evidence (55,73–75) suggests that TLS also occurs outside S-phase (and this work). Pol κ functions in NER in G1 as well as in quiescent cells (18,19). A recent report implicates also Pol η in mismatch repair (MMR) and shows that PCNA monoubiquitylation in G1 after oxidative stress is dependent on the MMR machinery in mammalian cells (17). These studies suggest that TLS polymerases can also function in G1 in a way coupled to DNA repair. Our observations further suggest that activation of CRL4^Cdt2^ on DNA damage in G1 is important to remove PIP degrons proteins thus facilitating Pol κ recruitment. CRL4^Cdt2^-mediated degradation of Cdt1 after DNA damage in G1 was recently shown to be dependent on the early incision steps of NER and subsequent PCNA loading (42,44,76), suggesting the existence of a time window when CRL4^Cdt2^ substrates compete out TLS polymerases for binding to PCNA during the early steps of DNA repair. This possibility is supported by our data obtained with Cdt1^R+4A^ mutant or a dominant negative mutant of Cullin 4A. Activation of CRL4^Cdt2^-mediated proteolysis may be a general mechanism that cells use to facilitate the interplay of specific repair factors on chromatin-bound PCNA after DNA damage, and be relevant to DNA repair by facilitating partners switch.

### Mechanism of interference of CRL4^Cdt2^ substrates with TLS polymerases

A detailed analysis of CRL4^Cdt2^ substrates has shown that a conserved TD motif confers high-affinity binding to PCNA (22), consistent with our findings that the threonine residue (T5) within the PIP degron of Cdt1 is required for both strong binding to PCNA and inhibition of Pol η focus formation. Canonical PIP boxes such as Fen1 lack the TD motif (Figure 5A). The p21 PIP box binds tightly to PCNA, with higher affinity than the PIP box of Fen1 (5), and we have shown that Cdt1 fails to impair Pol η focus formation when its PIP degron is replaced with the canonical PIP box of Fen1. Finally, introduction of a TD motif into canonical PIP boxes enhances binding to PCNA (22,23). Interestingly, the presence of a TD motif within the PIP box of PCNA-binding proteins is rare and it seems to be specifically present in CRL4^Cdt2^ substrates (Supplementary Figure S5A). Y-family DNA polymerase also have noncanonical PIP boxes (Supplementary Figure S5A) resulting in suboptimal PCNA-binding affinity (59). Consistent with this observation, both the PIP box and the ubiquitin-binding motifs are required for efficient Pol η accumulation to sites of UV damage (67). Hence, we propose that CRL4^Cdt2^ substrates interfere with recruitment of PCNA partners bearing noncanonical PIP boxes through an affinity-driven competition dependent on a highly conserved TD motif. At the molecular level, this model implies that PIP degrons should be able to mask the binding site for Pol η on PCNA (hydrophobic pocket). This hypothesis is supported by radiographic co-crystal structures (59,77) showing that Pol η PIP box motif interacts with PCNA on the same site bound by p21 (Supplementary Figure S5B).

In conclusion, the results presented in this work highlight the importance to remove PIP degrons from PCNA to facilitate TLS and probably other DNA repair mechanisms, such as NER and/or HR (70). Given their strong affinity for PCNA, failure to remove such factors may compromise DNA repair efficiency. Cdt1 is an oncogene (78) overexpressed in several human cancers and cancer cell lines (79,80). It will be interesting to determine if TLS function or DNA repair is affected in cancer cells overexpressing Cdt1, or other degrons, and if this may alter the sensitivity of cancer cells to specific chemotherapy.

## SUPPLEMENTARY DATA

Supplementary Data are available at NAR Online.

## FUNDING

The « Ligue Nationale Contre le Cancer » and the Ministry of Higher Education and Research [MNERT grant to N.T.]; Fellowship from la Fondation pour la Recherche Médicale (FRM) and Fondation ARC pour la Recherche sur le Cancer (to P.C.). Funding for open access charge: Centre National de la Recherche Scientifique (CNRS).

*Conflict of interest statement.* None declared.

## Supplementary Material

Supplementary Data
