# Supplementary material for: PIP degron proteins, substrates of CRL4Cdt2, and not PIP boxes, interfere with DNA polymerase η and κ focus formation on UV damage
Source: Nucleic Acids Res. 2014 Jan 14;42(6):3692–706. doi: 10.1093/nar/gkt1400 (PMC3973308; doi:10.1093/nar/gkt1400)
Supplement: Supplementary Data [file supp_42_6_3692__index.html]

PIP degron proteins, substrates of CRL4Cdt2, and not PIP boxes, interfere with DNA polymerase η and κ focus formation on UV damage — PIP degron proteins, substrates of CRL4Cdt2, and not PIP boxes, interfere with DNA polymerase η and κ focus formation on UV damage — Supplementary Data 

# PIP degron proteins, substrates of CRL4Cdt2, and not PIP boxes, interfere with DNA polymerase η and κ focus formation on UV damage

## Supplementary Data

files

**Files in this Data Supplement:**

- Supplementary Data - pdf file
