# Supplementary material for: PIP degron proteins, substrates of CRL4Cdt2, and not PIP boxes, interfere with DNA polymerase η and κ focus formation on UV damage
Source: Nucleic Acids Res. 2014 Jan 14;42(6):3692–706. doi: 10.1093/nar/gkt1400 (PMC3973308; doi:10.1093/nar/gkt1400)
Supplement: Supplementary Data [file supp_gkt1400_nar-02831-d-2013-File008.pdf]

PIP degron proteins, substrates of CRL4^{Cdt2}, and not PIP boxes interfere with DNA polymerase η and κ focus formation upon UV damage

Nikolay TSANOV, Chames KERMI, Philippe COULOMBE, Siem VAN DER LAAN, Dana HODROJ and Domenico MAIORANO

SUPPLEMENTARY MATERIALS AND METHODS

FACS analysis

Cells were co-transfected with pcDNA3-Cdt1-HA and pEGFP in the ratio of 10:1. Three days post-transfection, cells were harvested and fixed in 1% paraformaldehyde for 15 min at room temperature. After washing twice in PBS, cells were fixed in ice-cold 70% ethanol at -20°C overnight. Thawed cells were washed twice in PBS and incubated with 50 μg/mL RNase A at 37°C for 1h. DNA was stained with propidium iodide (25 μg/mL). Cells were analyzed with a FACScalibur flow cytometer using CellQuestPro software. Transfected cells were gated on the GFP signal.

Cyclin A staining

Cells expressing either Cdt1^{WT} or the Cdt1^{R+4A} mutant were transfected with either eGFP-Pol η or Pol κ- expressing plasmids. Twenty-four hours post-transfection cells were UV-irradiated. Following fixation cells were stained with an antibody specific for Cyclin A (sc-751, Santa Cruz Biothechnology) and analyzed by indirect immunofluorescence.

siRNA

The expression of Polk in NIH3T3 cells expressing the Cdt1^{R+4A} mutant was achieved as previously described (1). Total cell lysates were prepared 72 hours post-RNAi treatment and analyzed by western blot. The viability of cells was determined as described in Materials and Methods using the CellTiter-Glo® Luminescent Cell Viability assay (Promega).

SUPPLEMENTARY FIGURES LEGENDS

Supplementary Figure S1. Effects of CRL4^{Cdt2} inhibition on the relocalization of Pol η into nuclear foci after UV-induced DNA damage.

(*A*) U2OS cells transfected with eGFP-pol eta were pretreated with the proteasomal inhibitor MG132 for 1 hour prior to mock (-) or UV-irradiation (+). Control cells were treated with DMSO. After irradiation, cells were incubated for 4 hours in the presence of MG132 or DMSO, and the percentage of eGFP-Pol η-expressing cells displaying nuclear foci was assessed after fixation. Means and standard deviation of three independent experiments are shown.

(*B*) Schematic representation of mouse Cullin 4A wild-type (WT) and dominant-negative (dn) mutant constructs.

(*C*) UV-induced degradation of Cdt1 is inhibited by the dnCul4A mutant. NIH3T3 cells were transfected with the indicated constructs. After 24h, cells were mock (-) or UV- irradiated, and 4h later processed for immunoblotting with antibodies to myc, HA and β-actin.

(*D*) dnCul4A impairs the assembly of UV-induced Pol η foci. NIH3T3 cells were co-transfected with eGFP-Pol η and either dnCul4A-myc, or WT Cul4A-myc, or empty vector. Twenty-four hours post-transfection, cells were irradiated as in (*A*). Four hours later, the distribution of eGFP-Pol η in the nucleus was analyzed after fixation. Scale bar : 10μm.

(*E*) The percentage of eGFP- Pol η-expressing cells in which pol eta was localized in nuclear foci (shown in panel D) was assessed. Means and standard deviation of three independent experiments are shown.

Supplementary Figure S2. Cell cycle-dependent localization of eGFP-Pol κ.

(*A*) NIH-3T3 cells expressing either Cdt1^{WT} or the Cdt1^{R+4A} mutant of the experiment shown in Figure 1 were analyzed for eGFP-Pol κ nuclear foci formation and Cyclin A staining by immunofluorescence after fixation. Insets show selected cells (indicated by a white arrow) to appreciate formation of eGFP-Pol κ foci in Cyclin A-negative cells. Scale bar: 10 μM.

(*B*) The percentage of Cyclin A positive and negative cells in the total population of cells expressing either the empty vector, Cdt1 wild-type^{WT}, the Cdt1^{R+4A} or the Cdt1^{MutPIP} mutants and transfected with eGFP-Pol κ, was assessed after fixation. Means and standard deviation of three independent experiments are shown.

(C) Western blot of NIH3T3 cells expressing the Cdt1^{R+4A} mutant described in Figure 1 and treated with either a control siRNA (luciferase, Luc) or Pol κ-specific siRNA.

(D) Viability curves of NIH3T3 cells expressing the Cdt1^{R+4A} mutant treated with either a control siRNA (luciferase, Luc) or Pol κ-specific siRNA. NS, non-significative (n=3).

Supplementary Figure S3. Cdt1 interferes with UV-induced eGFP-Pol κ focus formation in a PIP box-dependent manner.

(*A*) NIH-3T3 cells were co-transfected with eGFP-Pol κ and either Cdt1^{WT}, Cdt1$^{\Delta PIP}$, or empty vector. Four hours after mock (-) or UV-irradiation (+), cells were fixed and stained with anti-HA. Scale bar: 10 μm.

(*B*) Quantification of the data shown in (*A*). Means and standard deviation of three independent experiments are shown.

(*C*) NIH-3T3 cells were co-transfected with eGFP-Pol κ and either myc-PIP box^{Cdt1WT}, myc-PIP box^{Cdt1Mut}, or empty vector and irradiated as in (*B*). Four hours later, the percentage of eGFP-Pol κ-expressing cells in which Pol κ was localized in nuclear foci was assessed. Means and standard deviation of three independent experiments are shown.

(*D*) Y-family DNA polymerases have non-canonical PIP boxes. Comparison of the PIP box sequences of Y-family DNA polymerases with that of the canonical PIP box (consensus).

Supplementary Figure S4. Cdt1 interference with eGFP-Pol η foci formation is independent of its function in DNA replication.

(*A*) Subcellular fractionation of NIH-3T3 cells transfected with wild-type Cdt1 (WT) or a Cdt1 mutant that lacks the PCNA-interaction motif (ΔPIP), or empty vector (ctrl.). Cells were mock (-) or UV-irradiated (+) 24 hours post transfection and 4h later were lysed and fractionated into soluble (cytoplasmic fraction) and insoluble (chromatin-bound) fraction as described in Materials and methods. Extracts were analyzed by western blot with the indicated antibodies. Activation of the DNA damage checkpoint was monitored by western blot with an anti-phospho-specific p53 antibody.

(*B*) Expression of Cdt1 at high levels in human U20S cells inhibits pol eta focus formation. U2OS cells co-transfected with eGFP-Pol η and either Cdt1^{WT} or empty vector were mock (-) or UV-irradiated (+). The percentage of eGFP-Pol η-expressing cells in which pol eta was localized in nuclear foci was assessed. Means and standard deviation of three independent experiments are shown.

(*C*) A functional PCNA-Interaction motif (PIP box) is required for the inhibition of pol eta focus formation by Cdt1. NIH3T3 cells were co-transfected with eGFP-Pol η and the indicated constructs. After 24 hours, cells were irradiated as in (*A*) and the percentage of cells displaying eGFP- Pol η foci was assessed. Means and standard deviation of three independent experiments are shown

(*D*) Overexpression of Cdt1 induces DNA rereplication in U2OS cells. Cells were transfected with the indicated Cdt1 constructs or empty vector (Vector). After 3 days, cells were fixed and stained with propidium iodide, and processed for FACS analysis. The extent of rereplication is indicated by the percent (%) of cells displaying DNA content higher than 4C.

(*E*) Cdt1 increases UV-induced cell death in a PIP box dependent fashion. NIH-3T3 cells were electroporated with either pcDNA3 vector encoding $Cdt1^{WT}$, or $Cdt1^{mutPIP}$, or empty vector. Twenty-four hours post-transfection, cells were mock- or UV-irradiated with 10 J/m^2. Cells were fixed and stained with propidium iodide 24 hours after irradiation, and processed for FACS analysis. The percentage of sub G1 cells was assessed.

Supplementary Figure S5. Conservation of the TD motif within PCNA-interacting proteins.

(*A*) Presence of the TD motif within PCNA-interacting motifs. Comparison of the sequences of the PIP box motifs of thirty known partners of PCNA in humans. The PIP boxes have been organized in several categories: Canonical PIP boxes (consensus), Non-canonical PIP boxes (differ from consensus), and PIP degrons (substrates of $CRL4^{Cdt2}$). The residues essential for optimal PCNA-interaction are shown in red and the residues that define the PIP degron are shown in blue. The proteins that contain a TD motif within their PIP box are indicated.

Ψ = Val, Leu, Ile, Met; ϑ = Tyr, Phe.

(*B*) Pol eta binds PCNA through a non-canonical PIP box onto the same hydrophobic pocket as p21. The images show the structures of PCNA-p21 complex (a) (2) and PCNA-pol eta complex (b) (3). The hydrophobic pocket onto PCNA was colored (orange: Pro 234, Pro 129, yellow: Tyr 250, light green: Leu 47, dark green: Ile 128). Pol eta binds PCNA through a non-canonical PIP box that lacks the glutamine residue (Q1), resulting in lower PCNA-binding affinity (3) . The positions of the TD motif and the R+4 residue in the structure of p21-PCNA are shown. The images were generated using PDB accession numbers 1AXC (a) and 2ZVK (b) and Swiss-PDB Viewer (4). The sequences of the PIP box motifs are indicated under the images.

SUPPLEMENTARY REFERENCES

1. Yoon, J.H., Bhatia, G., Prakash, S. and Prakash, L. (2010) Error-free replicative bypass of thymine glycol by the combined action of DNA polymerases kappa and zeta in human cells. *Proceedings of the National Academy of Sciences of the United States of America*, **107**, 14116-14121.
2. Gulbis, J.M., Kelman, Z., Hurwitz, J., O'Donnell, M. and Kuriyan, J. (1996) Structure of the C-terminal region of p21(WAF1/CIP1) complexed with human PCNA. *Cell*, **87**, 297-306.
3. Hishiki, A., Hashimoto, H., Hanafusa, T., Kamei, K., Ohashi, E., Shimizu, T., Ohmori, H. and Sato, M. (2009) Structural basis for novel interactions between human translesion synthesis polymerases and proliferating cell nuclear antigen. *J Biol Chem*, **284**, 10552-10560.
4. Guex, N. and Peitsch, M.C. (1997) SWISS-MODEL and the Swiss-PdbViewer: an environment for comparative protein modeling. *Electrophoresis*, **18**, 2714-2723.

Tsanov_Supplementary Figure S1

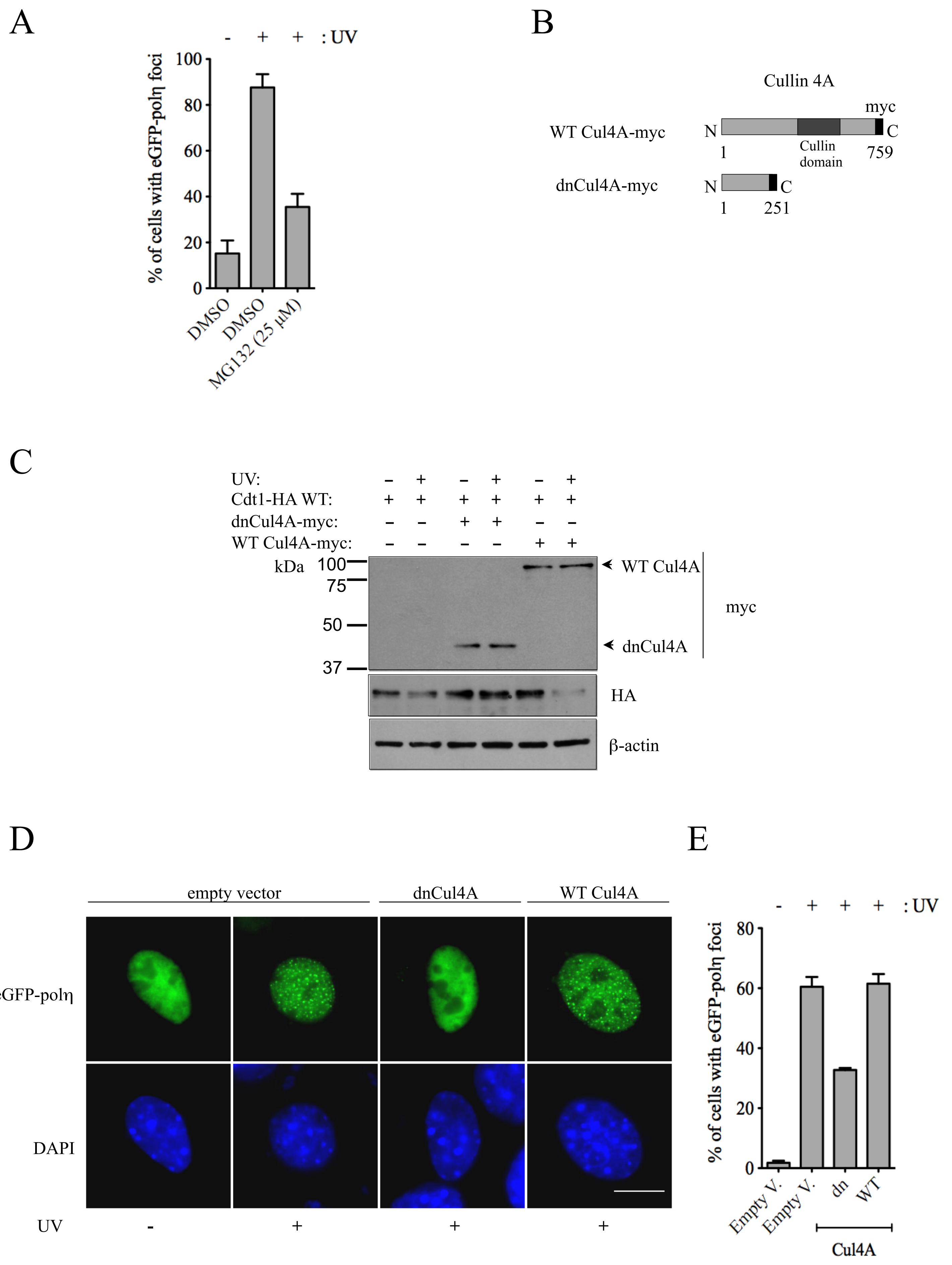

A

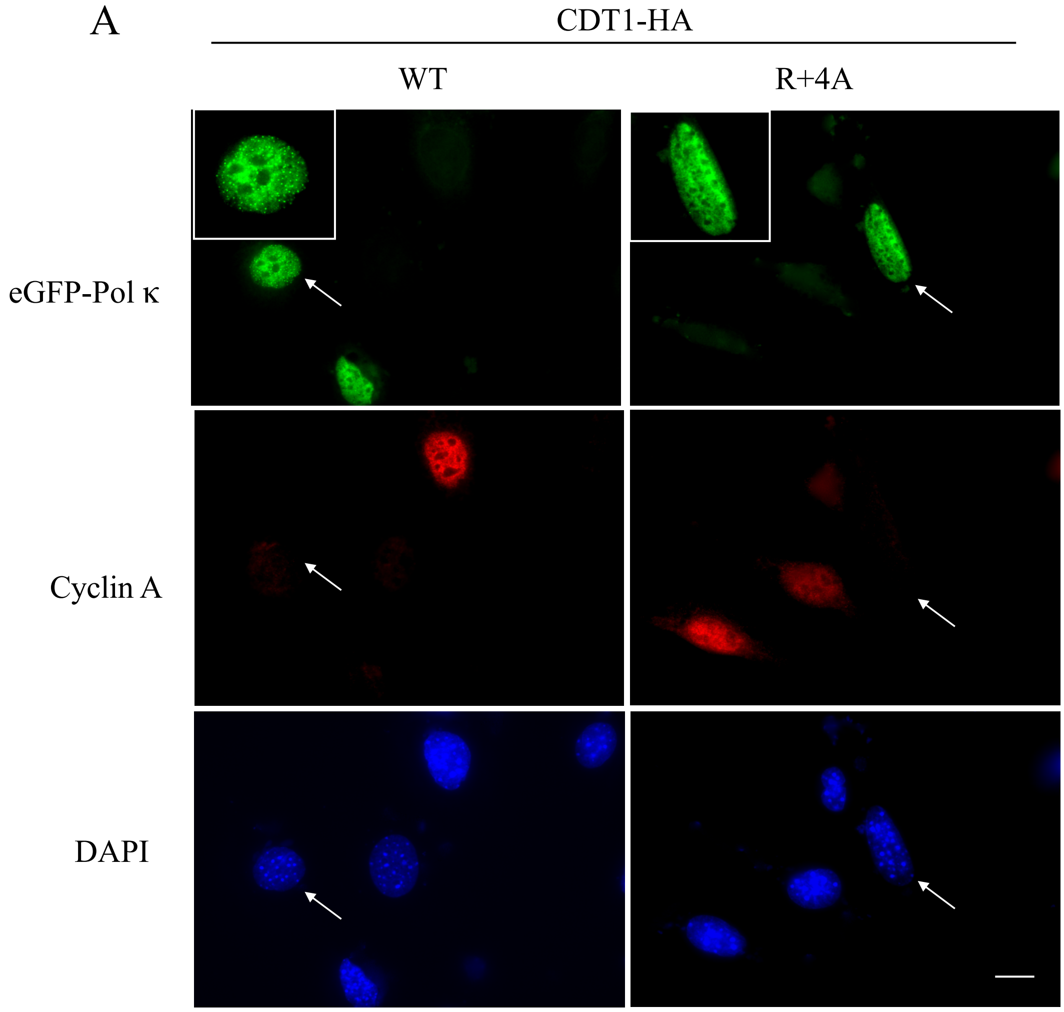
CDT1-HA
WT
R+4A
eGFP-Pol κ
Cyclin A
DAPI

B

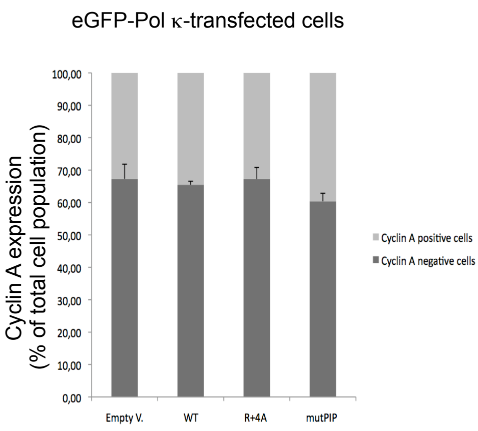
eGFP-Pol κ-transfected cells
Cyclin A expression
(% of total cell population)
100,00
90,00
80,00
70,00
60,00
50,00
40,00
30,00
20,00
10,00
0,00
Empty V.
WT
R+4A
mutPIP
Cyclin A positive cells
Cyclin A negative cells

C

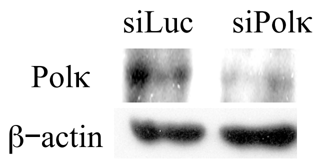
siLuc
siPolκ
Polκ
β−actin

D

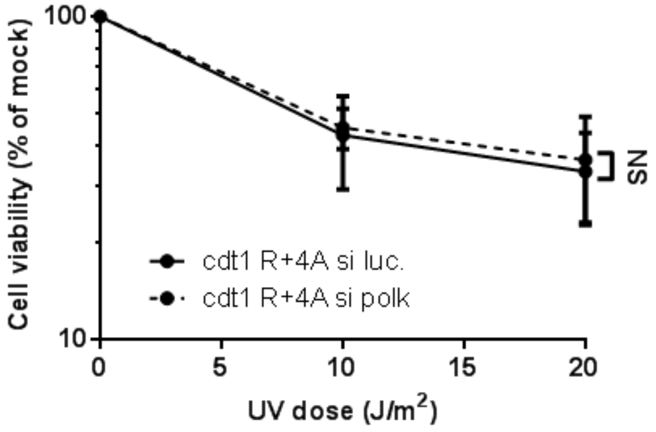
Cell viability (% of mock)
100
10
0
5
10
15
20
UV dose (J/m2)
NS
cdt1 R+4A si luc.
cdt1 R+4A si polk

A

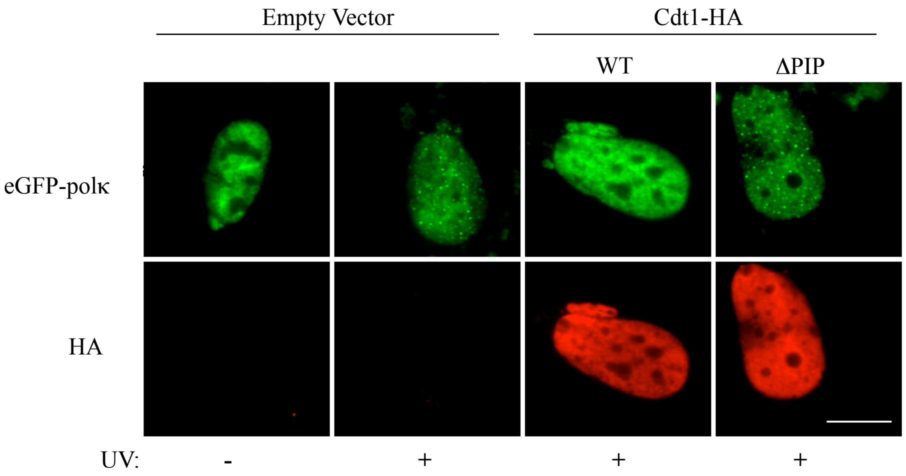

B

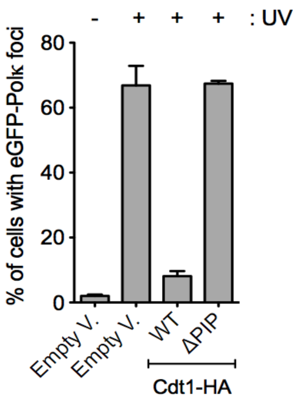

C

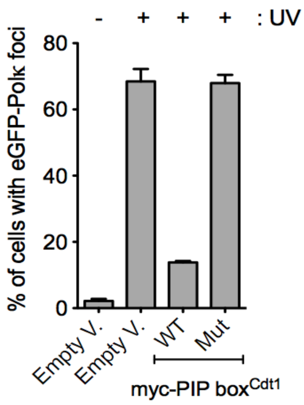

D

Canonical PIP box		12345678<br>QXXΨXXϑϑ
Y-family of DNA polymerases	polη	-MQTLESFFKPLT
	polκ	-KHTLDIFFK
	polι	-KGLIDYYL-
	Rev 1	PIP box absent

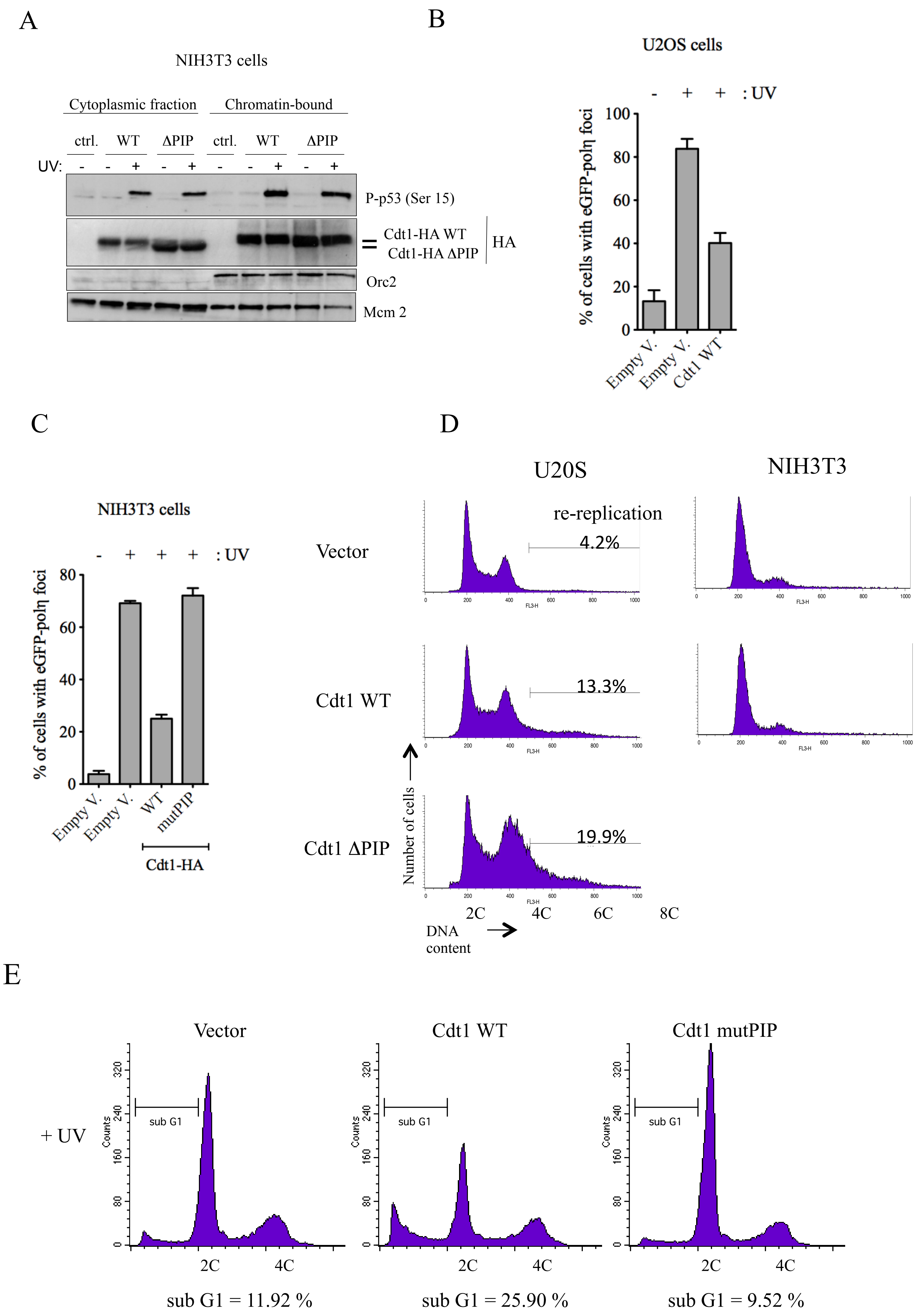
A
NIH3T3 cells
Cytoplasmic fraction
Chromatin-bound
ctrl.
WT
ΔPIP
ctrl.
WT
ΔPIP
UV: - - + - + - - + - +
P-p53 (Ser 15)
Cdt1-HA WT
Cdt1-HA ΔPIP
HA
Orc2
Mcm 2
B
U2OS cells
- + + : UV
% of cells with eGFP-polη foci
100
80
60
40
20
0
Empty V.
Empty V.
Cdt1 WT
C
NIH3T3 cells
- + + + : UV
% of cells with eGFP-polη foci
80
60
40
20
0
Empty V.
Empty V.
WT
mutPIP
Cdt1-HA
D
U20S
NIH3T3
re-replication
Vector
4.2%
Cdt1 WT
13.3%
Cdt1 ΔPIP
19.9%
Number of cells
2C
4C
6C
8C
DNA content
E
Vector
Cdt1 WT
Cdt1 mutPIP
+ UV
Counts
sub G1
2C
4C
sub G1 = 11.92 %
sub G1 = 25.90 %
sub G1 = 9.52 %

Tsanov_Supplementary Figure S5

A

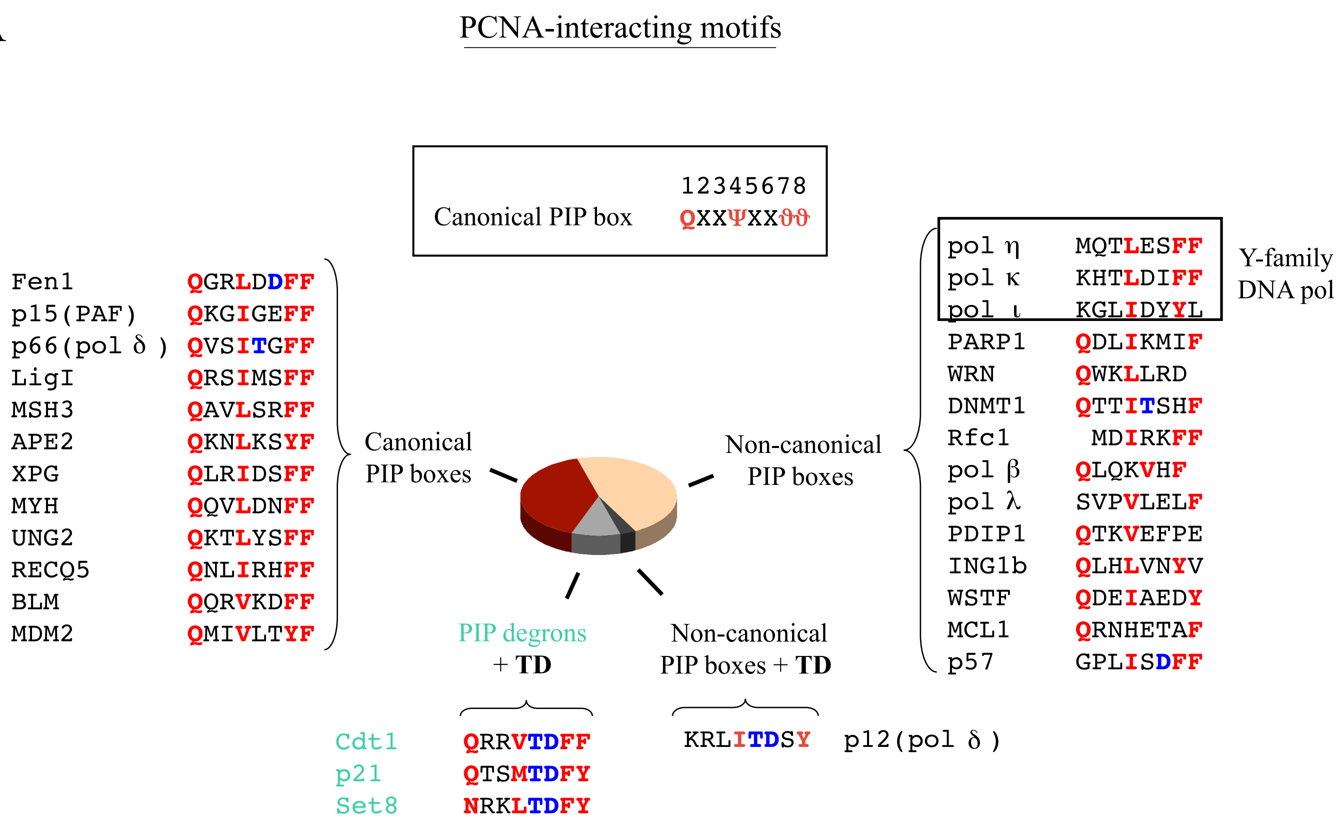

B

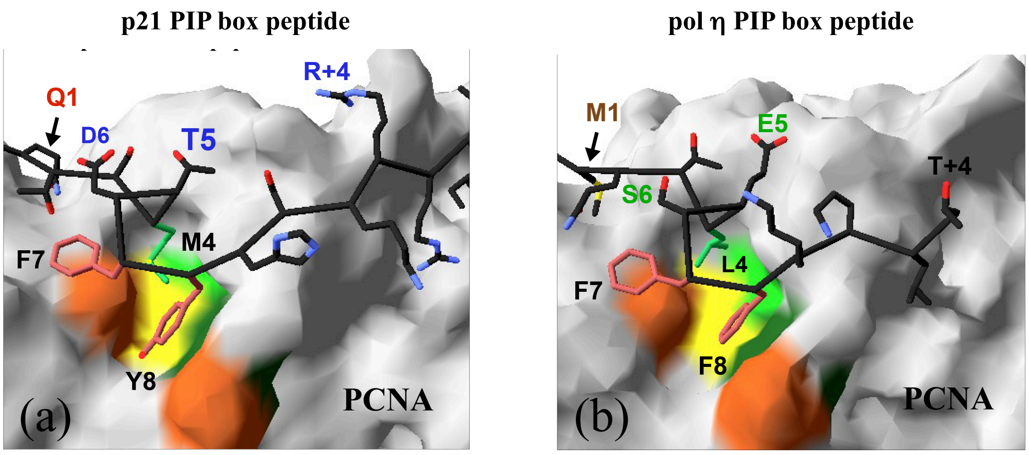

PIP box sequence : Q_1-X-X-M_4-T_5-D_6-F_7-Y_8-X-X-X-R_{+4} M_1-X-X-L_4-E_5-S_6-F_7-F_8-X-X-X-T_{+4}
